# Exploring the underlying mechanisms of fisetin in the treatment of hepatic insulin resistance via network pharmacology and in vitro validation

**DOI:** 10.1186/s12986-023-00770-z

**Published:** 2023-11-23

**Authors:** Tian Li, Junjun Ling, Xingrong Du, Siyu Zhang, Yan Yang, Liang Zhang

**Affiliations:** 1 Metabilic Vascular Disease Key Laboratory of Sichuan Province, Luzhou, 646000 China; 2 Drug Discovery Research Center, Southwest Medical University, Luzhou, 646000 China; 3 Affiliated Hospital of Guizhou Medical University, Guiyang, 550000 China; 4 Chongqing Tongnan NO.1 Middle School, Tongnan, 402660 China; 5 School of Medicine, University of Electronic Science and Technology of China, Chengdu, 610000 China

**Keywords:** Hepatic insulin resistance, Fisetin, Network pharmacology, Epidermal growth factor receptor, PI3K/AKT signaling

## Abstract

**Objective:**

To characterize potential mechanisms of fisetin on hepatic insulin resistance (IR) using network pharmacology and in vitro validation.

**Methods:**

Putative targets of fisetin were retrieved from the Traditional Chinese Medicine Systems Pharmacology database, whereas the potential genes of hepatic IR were obtained from GeneCards database. A protein–protein interaction (PPI) network was constructed according to the intersection targets of fisetin and hepatic IR using the Venn diagram. The biological functions and potential pathways related to genes were determined using Gene Ontology (GO) and Kyoto Encyclopedia of Genes and Genomes (KEGG) analyses. Cell experiments were also conducted to further verify the mechanism of fisetin on hepatic IR.

**Results:**

A total of 118 potential targets from fisetin were associated with hepatic IR. The areas of nodes and corresponding degree values of TP53, AKT1, TNF, IL6, CASP3, CTNNB1, JUN, SRC, epidermal growth factor receptor (EGFR), and HSP90AA1 were larger and could be easily found in the PPI network. Furthermore, GO analysis revealed that these key targets were significantly involved in multiple biological processes that participated in oxidative stress and serine/threonine kinase activity. KEGG enrichment analysis showed that the PI3K/AKT signaling pathway was a significant pathway involved in hepatic IR. Our in vitro results demonstrated that fisetin treatment increased the expressions of EGFR and IRS in HepG2 and L02 cells under normal or IR conditions. Western blot results revealed that p-AKT/AKT levels were significantly up-regulated, suggesting that fisetin was involved in the PI3K/AKT signaling pathway to regulate insulin signaling.

**Conclusion:**

We explored the pharmacological actions and the potential molecular mechanism of fisetin in treating hepatic IR from a holistic perspective. Our study lays a theoretical foundation for the development of fisetin for type 2 diabetes.

**Supplementary Information:**

The online version contains supplementary material available at 10.1186/s12986-023-00770-z.

## Introduction

Diabetes is a multifactorial chronic health condition that affects a large portion of the population, and 90–95% of patients with diabetes have type 2 diabetes (T2D) [1, 2]. In-depth investigations on T2D have identified insulin resistance (IR) as a core risk factor of T2D, making it a hot topic for scholars worldwide. In an insulin-resistant liver, common in T2D, abnormal liver insulin signaling pathway decreases liver glucose output and increases gluconeogenesis and glycogen decomposition, while lipid deposition in the liver further reduces insulin sensitivity, thus forming a vicious cycle [3]. Therefore, the influence of hepatic IR on the overall pathophysiological mechanism of metabolic diseases is significant, especially on the level of fasting blood glucose and basal insulin. However, so far, no effective drugs and methods to treat hepatic IR have been introduced, so it is essential to develop drugs that can effectively reverse IR.

**Fig. 1:**
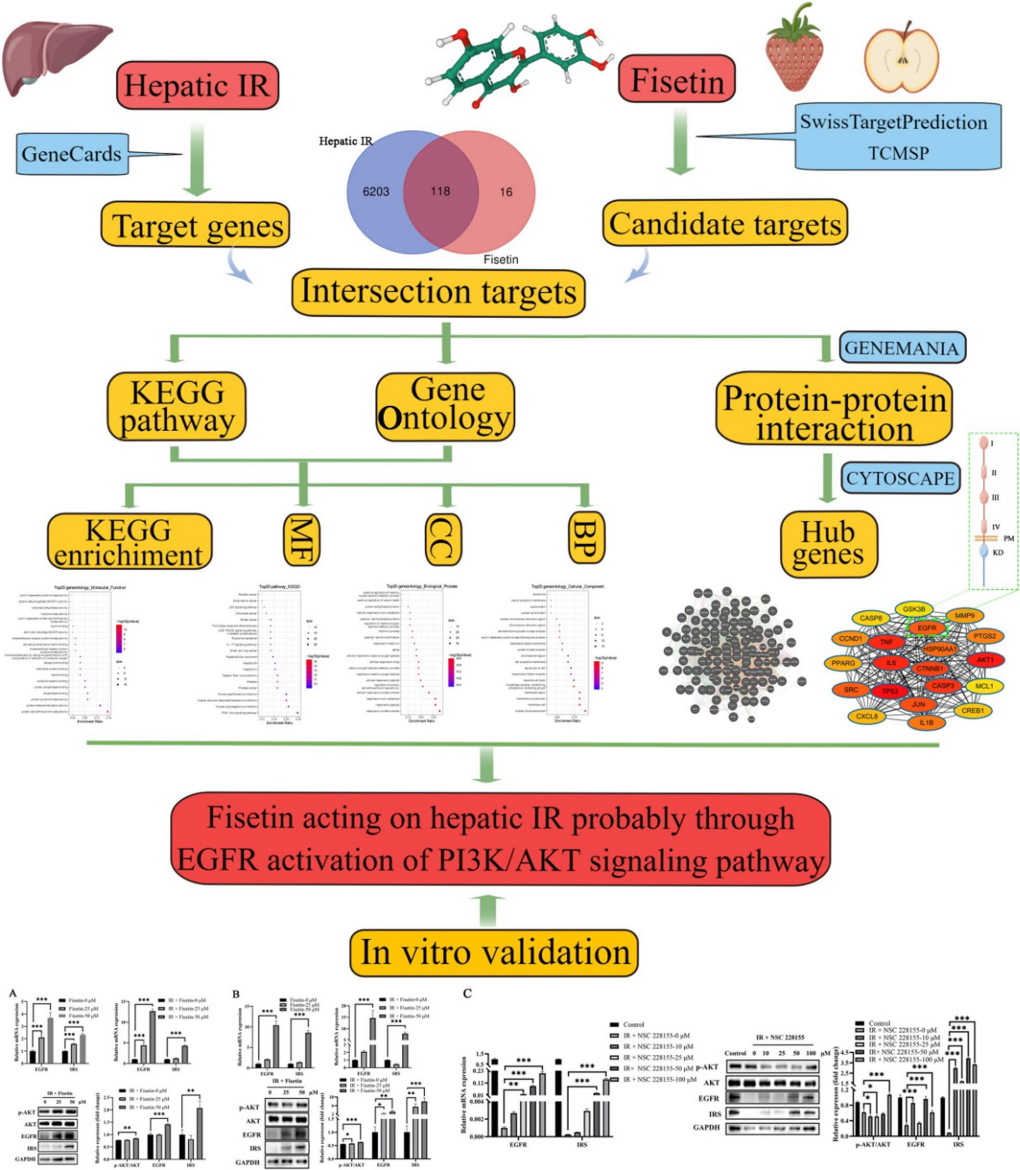
The design profile of the current study. Hepatic IR: hepatic insulin resistance; BP: biological process; CC: cellular component; MF: molecular function. The green box indicates Schematic structure of EGFR. On the left side of Fisetin is its chemical structure

Metabolic diseases are often diet-related and have a chronic course, and finding dietary components in fruits and vegetables as a way to prevent and treat chronic diseases is gaining widespread attention. Fisetin (3’, 3’, 4’, 7 – tetrahydroxyflavone, Fig. 1) is a flavonoid widely found in natural plants and is abundant in vegetables and fruits. In vivo and in vitro experiments have shown that fisetin exhibits a significant hypoglycemic effect [4]. Shen et al. evaluated the α-glucosidase inhibitory activity of fisetin extracted from Cotinus coggygria Scop [5]. In high-fructose-diet mice, fisetin was found to significantly inhibit the NF-κB pathway in activated B cells and activate the Nrf2 pathway, thereby reversing insulin resistance and liver injury [6]. Dietary fisetin supplements have been demonstrated to improve liver steatosis by limiting fat production and promoting lipolysis in the mouse liver. Moreover, research has shown that fisetin can reduce the production of excess reactive oxygen species by increasing extracellular matrix accumulation in liver, and reduce liver gluconeogenesis and proinflammatory response [7]. Therefore, fisetin is a natural dietary active substance that can improve hepatic IR owing to its non-toxic, safe, and efficient characteristics [8, 9]. Despite the above findings, the molecular mechanisms underlying the therapeutic effects of fisetin on hepatic IR remain unclear. Thus, further research with appropriate approaches is warranted to comprehensively elucidate the involved potential mechanisms.

With the development of bioinformatics and the support of high-throughput data analysis, network pharmacology is increasingly used in the study of potential targets and molecular mechanisms of the key materials in complex diseases. In this study, we used public database resources and computational tools to investigate the pharmacological network of fisetin in treating hepatic IR, including potential target proteins and pathways using network pharmacology, as well as the molecular mechanisms by in vitro validation.

## Materials and methods

### Candidate targets of fisetin

Traditional Chinese Medicine Systems Pharmacology (TCMSP) Database and Analysis Platform is a unique pharmacology platform of Chinese herbal medicine system and can elucidate drug targets at the systematic level based on the method of systematic pharmacology. The all target genes of fisetin were obtained from the TCMSP database. The targets were also predicted by SwissTargetPrediction database (http://www.swisstargetprediction.ch/) based on the similarity of two- or three-dimensional structure of fisetin from the PubChem website [10]. Then, candidate targets of fisetin were obtained by merging genes obtained in the two ways.

### Target genes of hepatic IR

Hepatic IR-related targets were obtained from GeneCards database (http://www.genecards.org) [11], a powerful database of human genetic information, providing comprehensive information on all annotations. Related target genes of this disease were obtained after inputting “liver insulin resistance” in the keyword column.

### Intersection targets of fisetin and hepatic IR

Through the Venn diagram, the target data sets of fisetin and hepatic IR were both uploaded, and the intersection was identified as the common gene targets, that is, the potential target genes of fisetin in treating hepatic IR.

### Protein–protein interaction network construction and analysis

The anchor protein–protein interaction (PPI) network is composed of different proteins interacting with each other that provide the direction for further exploration of gene function. Protein interactions of the intersection target genes were predicted between fisetin and hepatic IR with the STRING database (https://cn.string-db.org) [12] and InAct database (http://www.ebi.ac.uk/intact) [13]. Based on the conditions of species “Homo sapiens”, the minimum interaction threshold “highest confidence” (> 0.9) and network type “physical subnetwork” for STRING and the species “Homo sapiens” and type “protein” for InAct, the PPI network was constructed after inputting the above potential targets.

The obtained results were imported into the Cytoscape software, and the plugin CytoHubba was used to calculate and obtain the parameter values of the potential targets, such as degree, betweenness, closeness. The hub genes were screened and identified by the degree value.

### Gene ontology and KEGG pathway enrichment analyses

The Gene Ontology (GO) database includes three categories: biological process (BP), cellular component (CC), and molecular function (MF), which describes the possible molecular functions, the cellular environment, and the involved BPs of gene products. The above intersection targets of fisetin and hepatic IR were analyzed with R software package clusterProfiler (version 3.14.3), by setting the minimum gene set to 5, and the maximum gene set to 5000. The false discovery rate (FDR) < 0.05 was regarded as the screening condition for statistical difference.

The Kyoto Encyclopedia of Genes and Genomes (KEGG) database system was used to analyze the metabolic pathways of gene products in cells. The KEGG REST API was used to obtain recent KEGG pathway gene annotations as the background. The above intersection targets of fisetin and hepatic IR were analyzed with R software package clusterProfiler (version 3.14.3) after placing genes into the background sets. The parameter settings were the same as above.

### Pharmacological network construction

The component–target–disease network contributed to intuitively understand and analyze the potential combination of therapeutic mechanisms of components on multiple targets. The fisetin–target–hepatic IR network was constructed using the Cytoscape software. The “node” represented fisetin or hepatic IR or the intersection targets of fisetin and hepatic IR, while “edge” represented the interaction relationships.

### Chemicals and reagents

Fisetin (98%) was purchased from RHAWN (Shanghai, China). NSC228155 (EGFR activator) was purchased from Sigma Aldrich (MO, USA). Fisetin (50 mM) and NSC228155 (20 mg/mL) were prepared and stored in dimethyl sulfoxide (DMSO), and diluted with culture medium as required. The vehicle control in all experiments was 0.2% (v/v) DMSO. The antibody phospho-Akt Ser473, AKT, EGFR, IRS and GAPDH were purchased from CST (USA).

### Cell culture and treatment

Human hepatic cells (HepG2, L02) were obtained from Human Fenghui Biotechnology Co., Ltd and cultured in the DMEM medium (ScienCell Research Laboratories, California), supplemented with 10% fetal bovine serum (FBS) in a 37℃, 5% CO_2_ incubator. For IR, HepG2 and L02 were exposed to 5 × 10^−7^ mol/L insulin 24 h to induce cell IR [14]. To clarify the role of fisetin, normal cells or IR cells were treated with fisetin (25 and 50 μM) or NSC 228155 (10, 25, 50, and 100 μM) for 24 h. DMSO treatment was used as the vehicle control. After 24 h incubation, the cell pellet was washed with phosphate-buffered saline (PBS) and cells were harvested for further experiments.

### Quantitative real-time PCR

The RNA from the collected cells was extracted using TRIzol reagents (Invitrogen, Carlsbad, CA). The RNA samples were pretreated with deoxyribonuclease I (Invitrogen Life Technologies, USA) and cDNA was synthesized with SuperScript Kit (Invitrogen Life Technologies, USA). Quantitative RT-PCR was amplified using the miScript SYBR Green PCR kit (TaKaRa, Dalian, China) and ABI PRISM 7700 cycler (Applied Biosystems, Foster City, CA). Each sample was repeated with ribosomal 18S RNA as an internal control. Folding changes of gene expression were measured using the 2^−ΔΔCT^ method. The results were expressed as mean ± SE. See Additional file 3: Table S1 for the sequence of all primers.

### Western blotting

The culture plate was cleaned twice with cold phosphate-buffered solution, and the total proteins were obtained from a cell lysis buffer for western blotting and immunoprecipitation (Beyotime Biotechnology, China) that contained a cocktail of 1% (v/v) protease inhibitors. Then the proteins were separated by 7.5% SDS-PAGE and then electrophoretically transferred to Immobilon polyvinylidene fluoride (Merck Millipore, Australia). After blocked by 5% (m/v) skim milk at room temperature for 2 h, the PVDF membrane was incubated with phospho-Akt Ser473 (1: 1000), AKT (1: 1000), EGFR (1: 1000), IRS (1: 1000) or GAPDH (1: 1000) antibody at 4 °C overnight. The corresponding horseradish peroxidase was then coupled to the second antibody and incubated at room temperature for 1 h. The protein signal was enhanced with the Omni-ECL™ Femto Light chemiluminescence kit (Epizyme, China) and then visualized with the ChemiDoc XRS imaging system (Bio-rad, USA). Image J was used to quantify protein expression levels.

### Statistical analysis

All data are expressed as mean ± standard deviation. The GraphPad Prism 8.0 software was used for mapping analysis. Relative gene expression was calculated using the 2^−ΔΔCt^ method. An independent sample *t*-test or a one-way ANOVA were used to analyze the significance of the difference. A *P*-value < 0.05 (double-tailed) was considered statistically significant.

## Results

Fisetin can improve IR and has a significant hypoglycemic effect. In this study, the molecular targets and the possible mechanisms of fisetin in the treatment of hepatic IR were explored (Fig. 1). A total of 118 intersection targets were screened for fisetin associated with hepatic IR, which is significantly involved in multiple BPs that participate in oxidative stress and serine/threonine kinase activity. The top 10 hub genes containing AKT1 and EGFR were identified, and the PI3K/AKT signaling pathway was considered to be the significant pathway involved in hepatic IR. The results were further verified by in vitro assays.

### Potential target genes of fisetin in treating hepatic IR

**Fig. 2:**
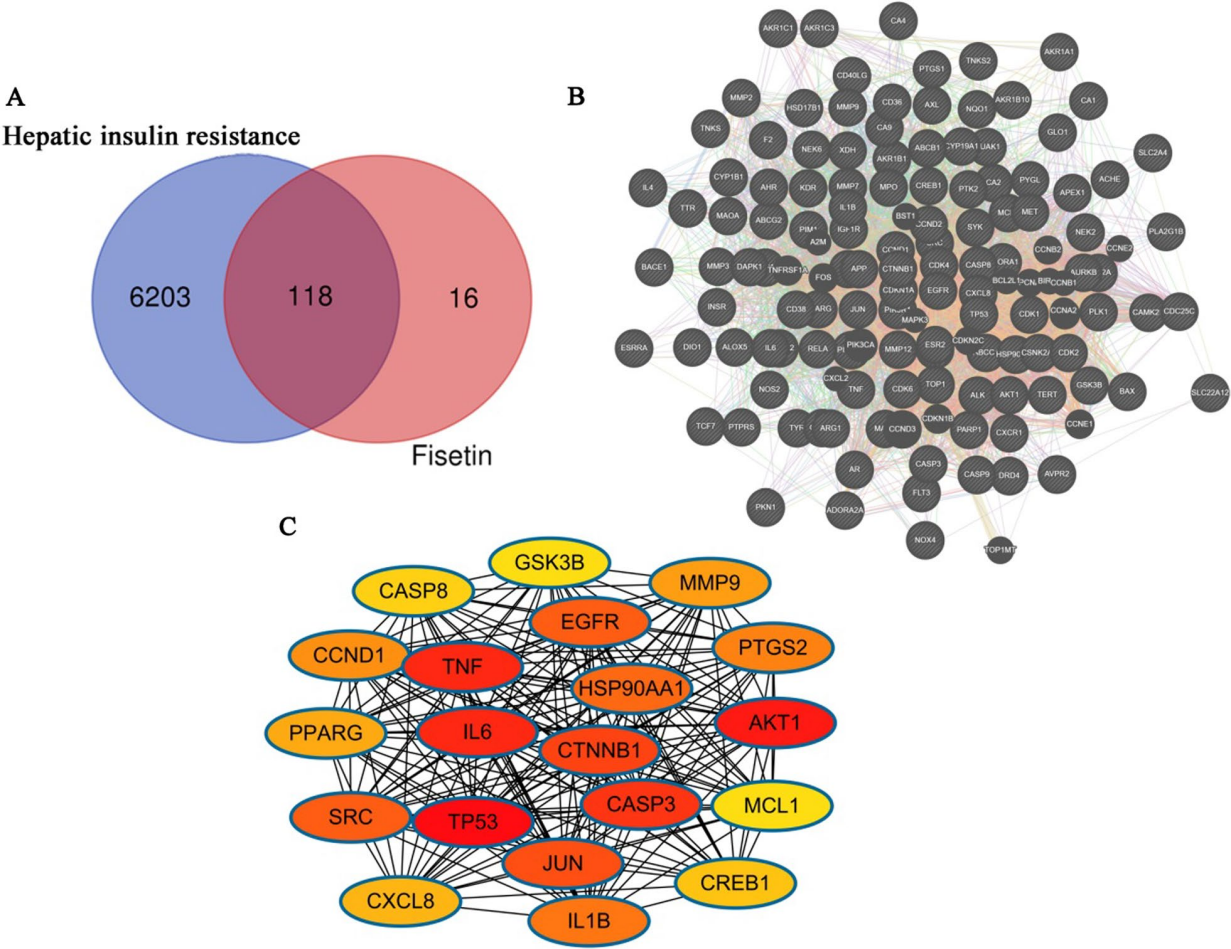
**A** Venny diagram of the fisetin targets and hepatic IR-related genes. **B** The PPI network of 118 intersection targets. **C** The top 20 hub gene networks of fisetin for hepatic IR treatment

The 134 targets of fisetin were obtained from the TCMSP database and the SwissTargetPrediction database. Based on the GeneCards database, a total of 6,321 targets associated with hepatic IR were obtained. Then, 118 intersection targets for the treatment of hepatic IR were identified by comparing the fisetin targets and the disease targets through the Venn diagram (Fig. 2A). These intersection targets may be the key proteins of fisetin in the treatment of hepatic IR.

### Construction of the PPI network regarding fisetin in treatment of hepatic IR

Network maps can be used to represent the type and intensity of interaction between coding genes during disease progression. Meanwhile, the extent to which a node contributes to the overall network can be ascertained by the number of connections between one node and other nodes, which is called degree. Hub genes are relatively high-degree genes, which have the strongest regulation effect on the differentially expressed gene set, playing a dominant role in the pathway, and are important targets.

To elucidate the molecular mechanism of fisetin on hepatic IR, the above 118 intersection targets were submitted to the STRING website to construct the PPI network (Fig. 2B). The PPI network consisted of 117 nodes, in which each node represented one protein, and each edge represented the protein–protein relationships, after hiding disconnected nodes. The average node degree and the average local clustering coefficient were 45.299 and 0.316, respectively. The top 20 hub genes, included TP53, AKT1, TNF, IL6, CASP3, CTNNB1, JUN, SRC, EGFR, HSP90AA1, IL1B, PTGS2, CCND1, MMP9, PPARG, CXCL8, CREB1, CASP8, MCL1, and GSK3B, which were considered potential hub targets of fisetin in treating hepatic IR and were identified based on the topological parameters (Fig. 2C) on the basis of three major network parameters of “betweenness”, “closeness”, and “degree”. The network characteristics of the 117 intersection targets are presented in Additional file 3: Table S2. To investigate the molecular mechanisms more precisely, we filtered for protein–protein interactions alone to construct another PPI network used by STRING (Additional file 1: Figure S1). Results showed that the above top 20 hub genes were found in STRING database of top 50 targets by the degree value, and 8 of them were in the top 10 targets ranked, including TP53, HSP90AA1, EGFR, SRC, CTNNB1, JUN, CASP3, MCL1 (Additional file 3: Table S3). Meanwhile, InAct is an open source, open data molecular interaction database populated with data either from literature curation or from direct data deposition. We also constructed another PPI using InAct and found that all above hub targets for direct physical interactions screened by STRING were included (Additional file 2: Figure S2 and Additional file 3: Table S4).

### GO and KEGG pathway enrichment analysis

**Fig. 3:**
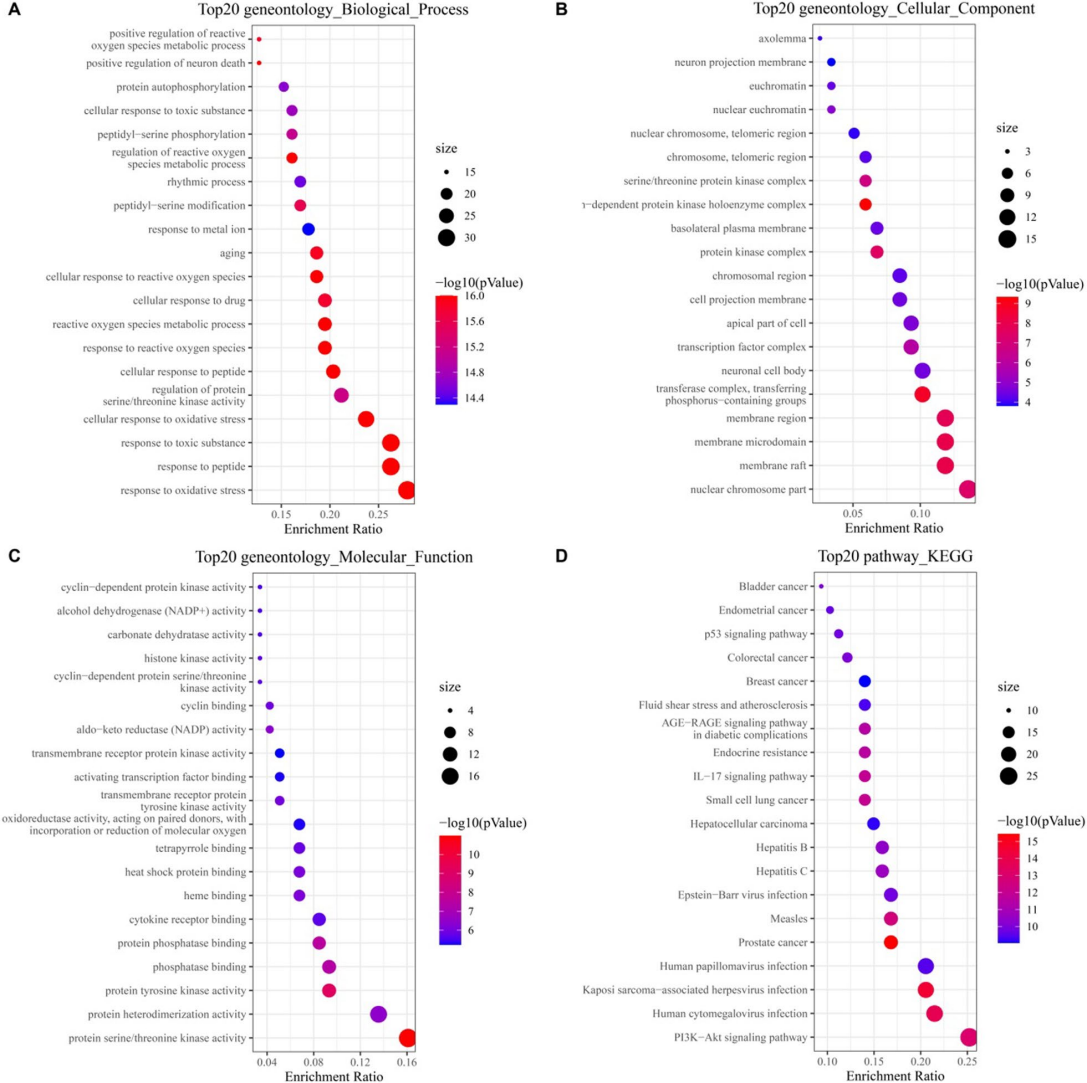
GO and KEGG analysis of the 118 intersection targets from fisetin were associated with hepatic IR. **A** GO biological process terms. **B** GO cellular component terms. **C** GO molecular function terms. **D** KEGG pathway. The size of each dot corresponds to the number of gene annotated in the entry, and the color of each dot corresponds to the corrected *p*-value

Next, GO enrichment analysis was performed to explore the multiple biological functions of putative targets of fisetin on hepatic IR. There were respectively 1,885 BPs, 68 CCs, and 153 MFs terms in total. The top 20 significantly enriched GO terms in BP, CC, and MF are plotted in Fig. 3, showing that the BPs were significantly enriched in the response to oxidative stress (GO: 0006979), peptide (GO: 1901652), and toxic substance (GO: 0009636), cellular response to oxidative stress (GO: 0034599), and regulation of protein serine/threonine kinase activity (GO: 0071900; Fig. 3A). CCs were significantly enriched in nuclear chromosome part (GO: 0044454), membrane raft (GO: 0045121), membrane microdomain (GO: 0098857), membrane region (GO: 0098589), transferase complex, transferring phosphorus-containing groups (GO: 0061695; Fig. 3B). Finally, MFs were significantly enriched in protein serine/threonine kinase activity (GO: 0004674), protein heterodimerization activity (GO: 0046982), chromatin binding (GO: 0003682), protein tyrosine kinase activity (GO: 0004713), and phosphatase binding (GO: 0019902; Fig. 3C). The top 10 BPs, CCs, and MFs are presented in Additional file 3: Table S5 according to the count. Moreover, GO enrichment was also analyzed with the above top 20 hub genes (Additional file 3: Table S6), and the results showed that consisted with the enrichment results of 118 intersection targets, the BPs were significantly enriched in the response to oxidative stress (GO: 0006979), cellular response to oxidative stress (GO: 0034599), and regulation of protein serine/threonine kinase activity (GO: 0071900; Fig. 4A). For the above top 20 hub genes, CCs were significantly enriched in membrane microdomain (GO: 0098857), membrane raft (GO: 0045121) and plasma membrane raft (GO: 0044853; Fig. 4B). Also, MFs were significantly enriched in protein serine/threonine kinase activity (GO: 0004674), and phosphatase binding (GO: 0019902; Fig. 4C).

**Fig. 4:**
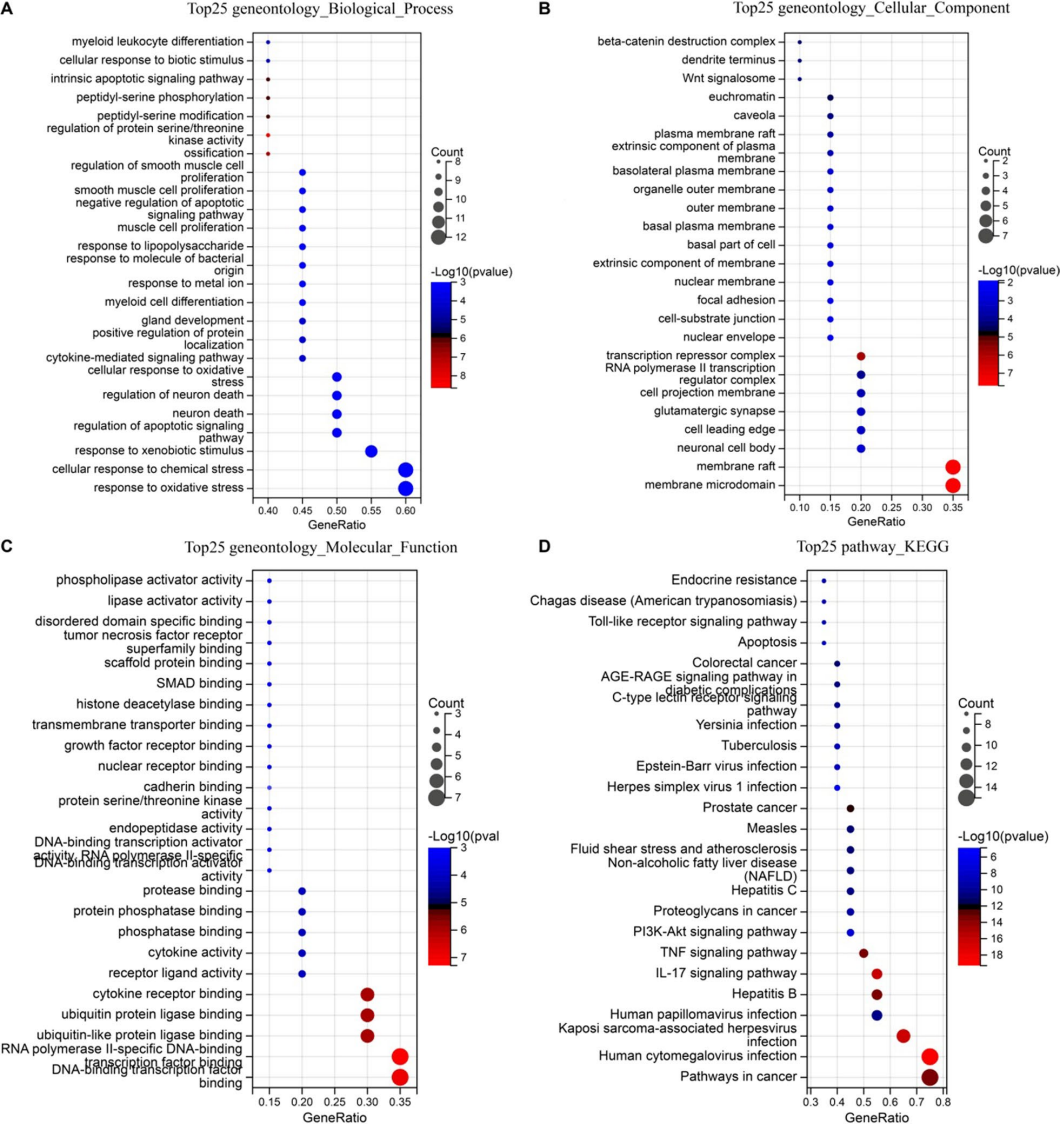
GO and KEGG analysis of the top 20 hub genes for fisetin in the treatment of hepatic IR. **A** GO biological process terms. **B** GO cellular component terms. **C** GO molecular function terms. **D** KEGG pathway. The size of each dot corresponds to the number of gene annotated in the entry, and the color of each dot corresponds to the corrected *p*-value

To further reveal the underlying mechanism on involved pathways of fisetin on hepatic IR, the 118 intersection targets and top 20 hub genes identified by the PPI network were conducted for KEGG pathway enrichment analysis. A total of 138 and 120 signaling pathways were acquired, and the 20 and 25 most significantly enriched pathways of fisetin on hepatic IR were ranked based on the parameters of counts as well as in combination with P-values, respectively (Additional file 3: Tables S7 and S8). The result showed that the PI3K/AKT signaling pathway (hsa04151) which was associated with the hepatic insulin signaling pathway, was enriched and may play a more critical role in the treatment of hepatic IR (Figs. 3D and 4D).

### Compound–target–disease network analysis

**Fig. 5:**
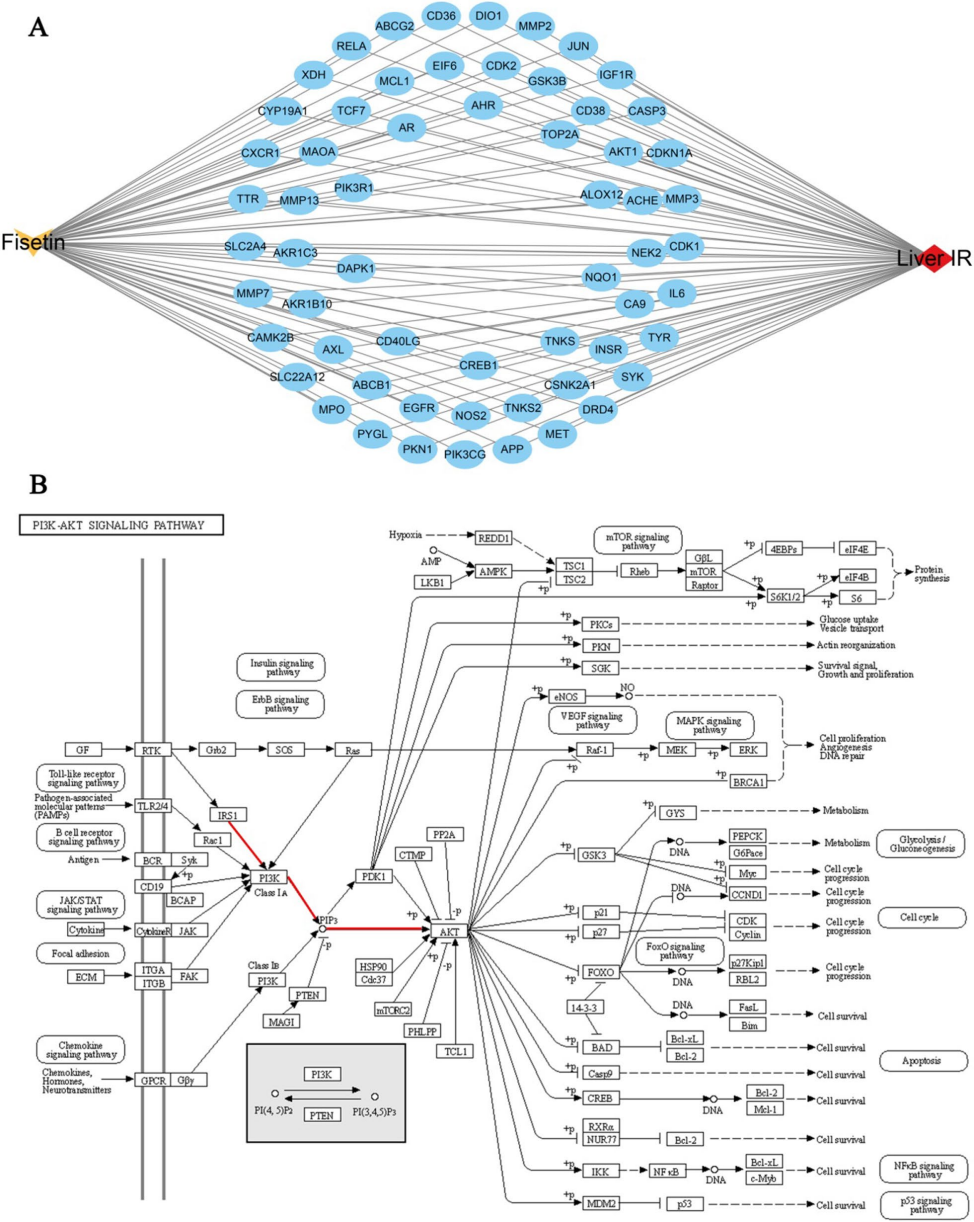
**A** The compound-targets-disease network. The yellow arrow represented fisetin, the red diamond represented hepatic IR, and blue circle represented the targets. **B** PI3K/AKT signaling pathway. The red lines show the possible signaling pathways through which fisetin acted on the hepatic IR

To clarify the molecular mechanism of fisetin alleviating hepatic IR, a compound–target–disease network was constructed based on top 60 hub genes of fisetin in treating hepatic IR (Fig. 5A). Particularly, PI3K/AKT signaling, which participates in canonical insulin signaling, was involved in the treatment of hepatic IR. The PI3K/AKT signaling pathway (map04151), as retrieved from the KEGG database (http://www.kegg.jp/kegg/mapper.html), is depicted in Fig. 5B.

### Functional analysis of insulin signaling by fisetin in vitro

**Fig. 6:**
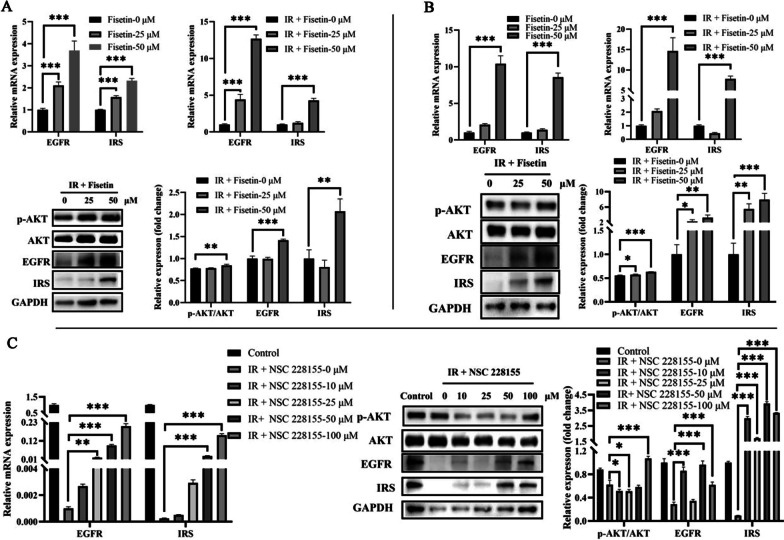
Effects of fisetin on EGFR and key factors (insulin receptor substrate (IRS) and AKT) in insulin signaling pathway in L02 and HepG2 cells. **A** Relative expression of RNA and proteins related to fisetin treatment in L02 normal or IR cells. **B** Relative expression of RNA and proteins related to fisetin treatment in HepG2 normal or IR cells. **C** Relative expression of RNA and proteins related to EGFR activator (NSC 228155) in L02 IR cells. Control represents untreated L02 cells. * *P* < 0.05, ** *P* < 0.01 and *** *P* < 0.001

Studies have been found that insulin receptor and EGFR share parallel activation mechanisms for signal transduction across the plasma membrane [15]. EGFR is a tyrosine kinase involved in cell proliferation, division, mitosis, and the occurrence of cancer and diabetes [16–18]. As one of the above 20 hub genes of fisetin acting on hepatic IR, EGFR (a typical transmembrane receptor, Fig. 1) is one of the few that activates multiple downstream effectors through ligand-induced dimerization-initiating signaling cascades that transmit extracellular signals into the cell [19]. So, whether fisetin can regulate insulin signaling pathway by activating EGFR? To test this hypothesis, the expressions of EGFR and key factors in insulin signaling pathway were detected in L02 cells after fisetin treatment. As shown in Fig. 6A, the EGFR and insulin receptor substrate (IRS) transcript levels were significantly up-regulated by fisetin at 25 and 50 μM in L02 cells. Western blot results showed that the levels of EGFR, IRS, and p-AKT/AKT were significantly increased after fisetin at 50 μM stimulation in L02 cells, which were incubated with insulin for 24 h to develop IR. These suggested that fisetin could activate EGFR and the key factors of insulin signaling pathways. Interestingly, in a state of IR, the stimulation of fisetin at 25 μM significantly increased EGFR expression, but had little effect on IRS expression in L02 cells. This implied that fisetin had a stronger regulatory effect on EGFR. Meanwhile, when IR-L02 cells were stimulated with EGFR activator, EGFR and IRS expressions were increased with the agonist dose, which was similar to the EGFR changes after fisetin stimulation (Fig. 6C). Likewise, EGFR and IRS expressions were also significantly up-regulated in normal or IR HepG2 cells which were considered to be a common IR model, under fisetin stimulation at 50 μM (Fig. 6B). Moreover, the level of p-AKT/AKT was positively correlated with dose of fisetin in HepG2 cells. These results suggested that fisetin increased the EGFR expression through IRS activating PI3K/AKT signaling pathway to alleviate hepatic IR.

## Discussion

For a long time, despite glucose-lowering therapy, 50% of the T2D patients require insulin therapy within 10 years. However, now to be sure, by restoring the normal metabolism of carbohydrates and fats, disease process can be reversed. Fisetin is a flavonoid ingredient as well as an antioxidant. It exists in fruits and vegetables, including strawberries, apples, and cucumbers. It has been found to reduce blood glucose levels in diabetic animals by enhancing glycolysis, inhibiting glycogen production and increasing glycogen storage [20]. It can also improve the damage of microvascular endothelial cells in diabetic brain [21]. In addition, fisetin plays an important role in improving inflammation, atherosclerosis, and tumor diseases [22–24]. In recent years, flavonoids have attracted more and more attention. Choi et al. found that flavonoids fisetin may help improve obesity, liver fibrosis and IR caused by high fat diet. Fisetin has been reported to be a potential agent for developing new strategies for diabetes treatment by inhibiting oxidative stress in human monocytes under conditions of hyperglycemia. However, the molecular mechanism of fisetin in the treatment of diseases is limited, and in-depth systematic studies are required to understand it at the molecular level. Using network pharmacology to explore the target of fisetin and its relationship with disease from a holistic perspective can provide a new strategy for studying its potential pharmacological mechanism.

Insulin resistance and β-cell dysfunction are the main pathophysiological factors leading to T2D. Insulin binds to the insulin receptor (a receptor tyrosine kinase, INSR), which leads to phosphorylation of IRS, which activates PI3K and AKT and further activates downstream signaling involving the AKT substrate [25]. However, the exact underlying causes of IR have not been fully elucidated, although many major mechanisms of IR have been proposed, including oxidative stress, inflammation, insulin receptor mutations, endoplasmic reticulum stress, and mitochondrial dysfunction [26]. In recent years, the liver has become an important organ to regulate IR. The liver regulates insulin sensitivity primarily through the metabolism of carbohydrates and fats, which control the body’s energy requirements. The results of our analysis of the network of intersection targets of fisetin on hepatic IR revealed that fisetin acted on 20 hub genes of hepatic IR based on degree values. However, some researchers have suggested that “betweenness” could be another critical trait in the selection of potential targets in network analysis [27]. Betweenness, or “bottleneckness,” is a key link-protein that has amazing functionality and dynamic characteristics. It measures the number of shortest paths through a certain node [28]. In this way, the top 10 hub genes of hepatic IR are screened out, including TP53, AKT1, CTNNB1, TNF, IL6, SRC, HSP90AA1, EGFR, CASP3, PTGS2A, which is highly repetitive with the results obtained with degree values. Among these hub genes, many were found to be involved in IR. AKT1 is a critical mediator of insulin/phosphatidylinositol 3-kinase signaling pathway [29]. TNF and IL6 are common inflammatory cytokines derived from visceral fat [30]. SRC is a non-receptor tyrosine kinase that regulates a wide range of cellular functions. It is involved in hepatocyte swelling by insulin, which contributes to p38 activation, leading to the inhibition of autophagic proteolysis [31, 32]. It has been found that insulin in isolated rat and mouse hepatocytes can trigger EGFR activation and hepatocyte proliferation [33], implying that EGFR is associated with insulin signaling pathways. Therefore, these signaling pathways play different functions in the development of hepatic insulin resistance, and fisetin may play an intervention role by regulating these signaling pathways.

GO pathway enrichment analysis revealed that the key intersection targets of IR affected by fisetin were primarily involved in BPs such as oxidative stress and serine/threonine kinase activity, and the location of action was concentrated on the cell membrane. Based on the known molecular mechanism of insulin signaling pathway, we speculated that fisetin may act on proteins located on the cell membrane with similar functions as INSR, thereby activating the downstream insulin metabolic pathway. Among the 20 hub genes screened above, EGFR, as a potential target, is another important central gene in cell membranes, besides INSR, according to bioinformatics analysis based on validated related genes in public disease gene banks [34, 35]. It has long been reported that EGFR and INSR may share a common evolutionary origin, and the extracellular domains of both contain structurally similar cysteine-rich regions and highly conserved specific tyrosine protein kinase domains [15, 36]. Studies have shown that loss of function of tyrosine protein kinase in EGFR pathway or abnormal activity or cell localization of key factors in the pathway can lead to the occurrence of tumor, diabetes, and immune deficiency [37–39]. Insulin in isolated rat and mouse hepatocytes can trigger EGFR activation and hepatocyte proliferation [33], suggesting that EGFR can regulate insulin signaling. The expression of EGFR in T2D patients was analyzed by enzyme-linked immunosorbent assay, which showed that EGFR was highly expressed in liver, but its expression was low in skeletal muscle and adipose tissue, suggesting that soluble EGFR in liver cells was associated with hepatic IR. Furthermore, EGFR levels were increased in serum in a diabetic mouse model and in 27 T2D patients. It is concluded that EGFR is a potential biomarker for evaluating IR in mouse and human serum [40]. Moreover, our previous study has discovered that EGFR and fisetin could effectively bind with each other through molecular docking studies [41]. But the limitation of the fisetin–target–hepatic IR network is that it is not possible to assess how fisetin regulates target genes such as EGFR. Therefore, in our study, it was confirmed that fisetin, like EGFR activator, could up-regulate the RNA and protein expressions of EGFR and IRS in HepG2 and L02 cells through cell experiments. Furthermore, fisetin continued to respond to EGFR and IRS by increasing the protein expressions in IR cell models. These results speculated that fisetin may activate EGFR tyrosine phosphorylation activity to enhance insulin-mediated downstream signal transduction. In particular, the up-regulation effect of fisetin on EGFR expression was stronger in liver cancer cells (HepG2), and the RNA expression was increased by about 10 times, while the expression of EGFR in normal liver cells (L02) was only increased by 3.6-fold under fisetin stimulation. Fisetin has proven its anti-cancer role in various cell types, such as colon, stomach, prostate and liver cancers [24, 42–44]. It has been proven that fisetin significantly enhanced the levels of E-cadherin in EGF-treated prostate cancer cells [43]. In this study, the strong activation of EGFR by fisetin in cancer cells might be a vital therapeutic approach for liver cancer treatment associated with the EGFR.

As observed in the results of KEGG pathway enrichment analysis, the key intersection targets of fisetin acting on hepatic IR was primarily related to the PI3K/AKT signaling pathway. Studies have shown that AKT, as a key protein kinase, is activated by a cascade of the main classical insulin signal required to maintain blood sugar levels in the liver. The AKT kinase is required for insulin regulation of pathways that controls glucose homeostasis throughout the body, including glucose transport in fat cells and muscle, liver glucose gluconeogenesis, and cellular autonomic activation of liver fat production [45]. On the one hand, AKT controls FOXO1 transcription in glucose in the liver, leading to phosphorylation and inhibition of FOXO1 function, resulting in the retention of FOXO1 in the cytoplasm and inactivation. On the other hand, insulin-activated AKT in the liver also strongly regulates lipid metabolism, primarily through the SREBP-1c transcription program. Thus, targeting this pathway-associated protein can activate downstream signaling responses during IR by phosphorylation by AKT [46]. Researchers have determined that phospholipase Cr1 interacts with activated EGFR and transactivates through PI3K-PKCβ1-AKT signaling in mesangial cells to mediate hyperglycemia-induced up-regulation of type I collagen, thereby affecting the development of diabetic nephropathy [47]. Therefore, it could be considered that the treatment of hepatic IR by fisetin mainly regulates EGFR by PI3K/AKT signaling pathway. In vitro experiments also validated that fisetin could significantly up-regulate the p-AKT/AKT levels in IR-L02 and IR-HepG2 cells and reduce the duration of cells with IR. The limitation of this study is that the mechanism of action of fisetin in regulating AKT downstream of insulin signaling was not investigated. Another noteworthy enrichment pathway is the AGE-RAGE signaling pathway (hsa04933) in diabetic pathway with small counts. Advanced glycation end product receptor (RAGE) is a receptor for advanced glycation end products involved in the development of diabetes-induced complications. It is involved in diabetes-induced hepatocyte apoptosis and increased inflammation [48]. In animal models, a positive correlation was observed between dietary AGE/glucose oxidation products and insulin sensitivity [49]. It has been reported that AGE-RAGE interactions cause fat to build up in the liver, leading to inflammation, fibrosis, IR, and other complications of fatty liver disease [50]. AGEs can induce the generation of RAGE, leading to the initiation of different internal signaling pathways, including PKC, PI3K/AKT, JAK/STAT, MAPK/ERK [51–53], leading to oxidative stress and chronic inflammation and aggravating further insulin resistance [54, 55]. The immune system, especially macrophages, has an important defense mechanism against RAGE pathway activity [50]. Therefore, there is another possible speculation on the mechanism of fisetin’s reversal of hepatic IR: fisetin reduces hepatic IR by regulating the AGE-RAGE signaling pathway to inhibit oxidative stress and chronic inflammation in the liver. In addition, many other diseases are enriched, which may be ascribed to the presence of the same molecular targets in different pathological processes of diseases, which is also a limitation of network pharmacology.

The etiology of IR is complex and multifaceted, involving cellular autonomic mechanisms and interorgan communication. IR in the liver can be traced to defects in insulin receptor levels and may therefore affect all branches of insulin signaling in hepatocytes. This study provides a network pharmacological method to explore the potential molecular mechanism of fisetin in treating hepatic IR. We conclude that fisetin could regulate insulin metabolism signaling pathway through EGFR then IRS activation of the PI3K/AKT signaling pathway or inhibiting oxidative stress and chronic inflammation by AGE-RAGE signaling pathway. Our study lays a theoretical foundation for the development of fisetin for T2D.

### Supplementary Information

**Additional file 1: Fig. S1** The PPI network of 118 intersection targets with STRING web tool using protein-protein interactions alone. The red lines represent experimentally determined, the blue lines represent from curated databases, and the yellow lines represent textming

**Additional file 2: Fig. S2** The PPI network of 118 intersection targets with InAct

**Additional file 3:** Supplemental Table 1-8.

## Data Availability

The datasets used and analyzed in this study are available from the corresponding author upon reasonable request.

## Abbreviations

IR: Insulin resistance
PPI: Protein–protein interaction
KEGG: Kyoto encyclopedia of genes and genomes
GO: Gene ontology
EGFR: Epidermal growth factor receptor
T2D: Type 2 diabetes
TCMSP: Traditional Chinese medicine systems pharmacology
BP: Biological process
CC: Cellular component
MF: Molecular function
FDR: False discovery rate
FBS: Fetal bovine serum
IRS: Insulin receptor substrate
INSR: Insulin receptor
