# Supplementary figures and images for: Exploring the underlying mechanisms of fisetin in the treatment of hepatic insulin resistance via network pharmacology and in vitro validation
Source: Nutr Metab (Lond). 2023 Nov 23;20:51. doi: 10.1186/s12986-023-00770-z (PMC10666360; doi:10.1186/s12986-023-00770-z)

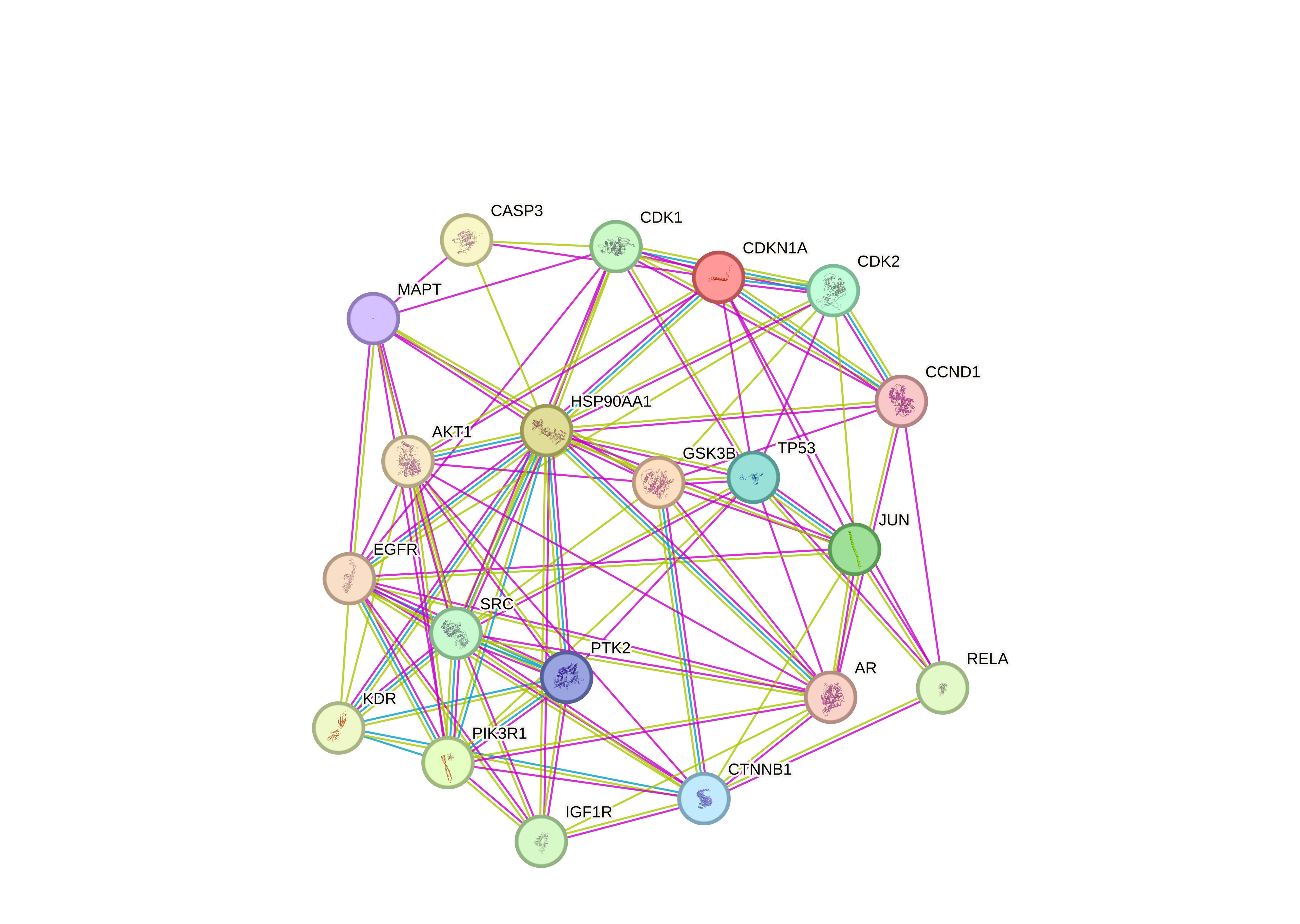

Supplement: Supplementary file 1 — Additional file 1: Fig. S1 The PPI network of 118 intersection targets with STRING web tool using protein-protein interactions alone. The red lines represent experimentally determined, the blue lines represent from curated databases, and the yellow lines represent textming [file 12986_2023_770_MOESM1_ESM.jpg]

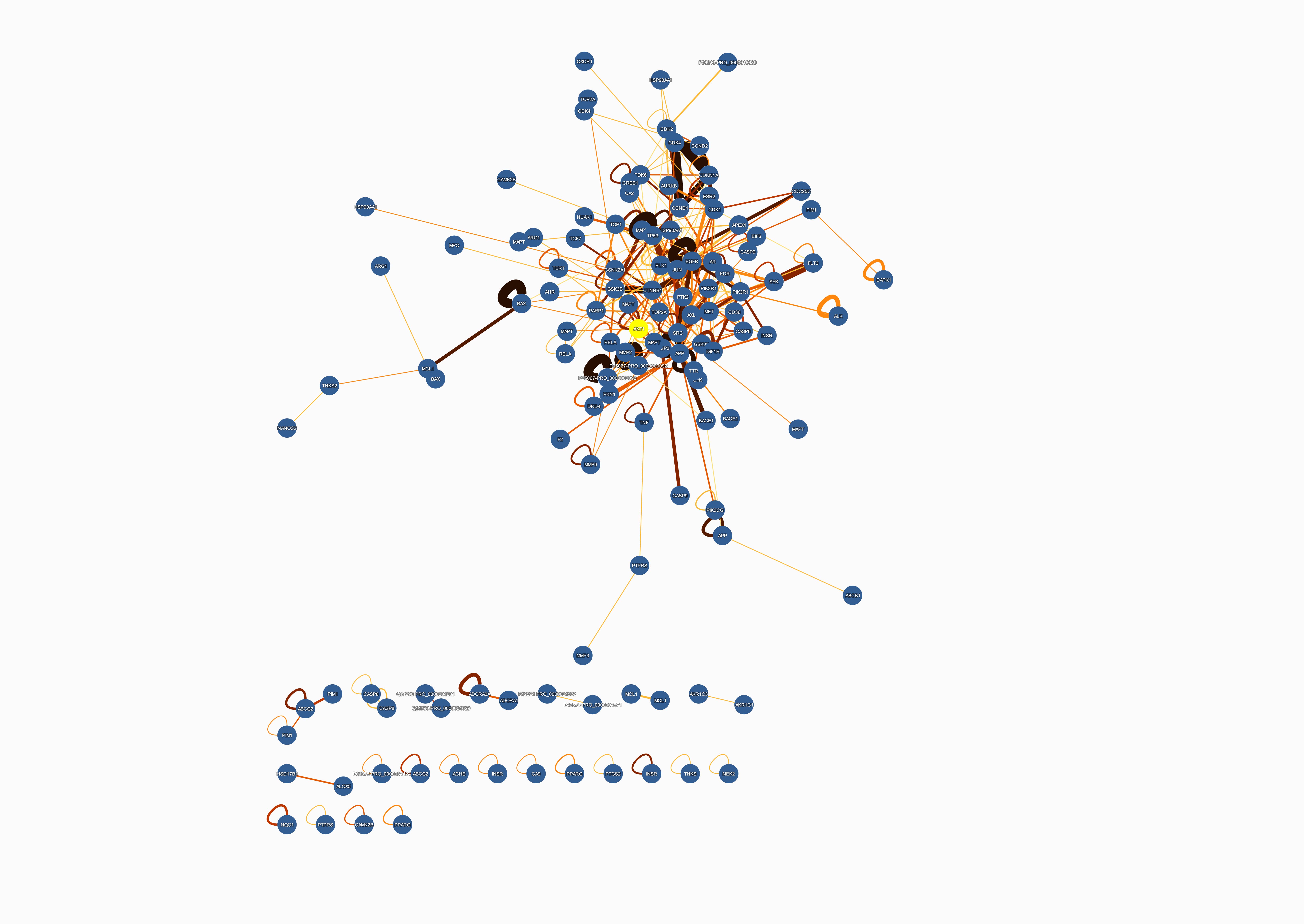

Supplement: Supplementary file 2 — Additional file 2: Fig. S2 The PPI network of 118 intersection targets with InAct [file 12986_2023_770_MOESM2_ESM.jpg]
