# Supplementary material for: Exploring the underlying mechanisms of fisetin in the treatment of hepatic insulin resistance via network pharmacology and in vitro validation
Source: Nutr Metab (Lond). 2023 Nov 23;20:51. doi: 10.1186/s12986-023-00770-z (PMC10666360; doi:10.1186/s12986-023-00770-z)
Supplement: Supplementary file 3 — Additional file 3: Supplemental Table 1-8. [file 12986_2023_770_MOESM3_ESM.docx]

**Supplemental Table 1. Primer sequences of qRT-PCR**

| Gene | Sequences (5’-3’) |
| --- | --- |
| EGFR | F: GCTATGAGATGGAGGAAGACGG  R: GAGATCGCCACTGATGGAGG |
| IRS | F: ATTTAAGCGCCTATGCCAGC  R: AGGATTTGCTGAGGTCATTTAGGT |
| 18s | F: CGGCTACCACATCCAAGGAA  R: GCTGGAATTACCGCGGCT |

**Supplemental Table 2. The PPI network characteristics of the 117 intersection targets with the STRING web tool.**

| Target | Betweenness | Closeness | Degree |
| --- | --- | --- | --- |
| TP53 | 1089.66093 | 96.33333 | 154 |
| AKT1 | 928.96629 | 95.66667 | 512 |
| TNF | 708.23114 | 92.83333 | 140 |
| IL6 | 695.98136 | 92.83333 | 140 |
| CASP3 | 526.33591 | 91 | 134 |
| CTNNB1 | 832.43163 | 90.33333 | 132 |
| JUN | 363.70217 | 88.16667 | 122 |
| SRC | 665.05372 | 87 | 120 |
| EGFR | 528.95867 | 87.66667 | 120 |
| HSP90AA1 | 575.62134 | 87 | 118 |
| IL1B | 337.78249 | 86 | 114 |
| PTGS2 | 436.32343 | 85.5 | 112 |
| CCND1 | 256.98582 | 84 | 108 |
| MMP9 | 188.93981 | 81.83333 | 100 |
| PPARG | 341.14904 | 82.16667 | 98 |
| CXCL8 | 304.45855 | 79.5 | 92 |
| CREB1 | 220.93372 | 79.16667 | 88 |
| CASP8 | 93.98197 | 7.16667 | 86 |
| MCL1 | 74.12843 | 76.5 | 82 |
| GSK3B | 121.57695 | 77 | 82 |
| CDKN1A | 66.47979 | 76.16667 | 80 |
| IL4 | 266.71611 | 75.66667 | 76 |
| MMP2 | 33.46734 | 75 | 74 |
| CDK4 | 60.71308 | 74.16667 | 72 |
| RELA | 39.02707 | 74.66667 | 72 |
| AR | 183.57777 | 74.33333 | 70 |
| PIK3R1 | 56.11658 | 73.5 | 68 |
| PARP1 | 142.67862 | 73.66667 | 68 |
| IGF1R | 74.12883 | 73.33333 | 68 |
| CDK2 | 38.87421 | 72.66667 | 66 |
| CASP9 | 17.26993 | 72.5 | 66 |
| CDK1 | 77.66514 | 71.5 | 62 |
| KDR | 38.73398 | 71.83333 | 62 |
| APP | 170.56363 | 71.16667 | 58 |
| MET | 26.97067 | 70.5 | 56 |
| CDK6 | 17.19227 | 69.5 | 54 |
| MPO | 54.83058 | 69.16667 | 54 |
| TERT | 44.46899 | 68.66667 | 52 |
| PTK2 | 61.18382 | 68.66667 | 50 |
| ESR2 | 161.5988 | 68.83333 | 50 |
| CDC25C | 75.2359 | 67.16667 | 48 |
| SYK | 31.4454 | 67.83333 | 46 |
| CYP19A1 | 82.70018 | 68 | 46 |
| MAPT | 136.59811 | 67.5 | 46 |
| AHR | 247.57965 | 68.66667 | 46 |
| CD40LG | 15.27119 | 66.16667 | 44 |
| NOS2 | 13.24365 | 67 | 44 |
| CCND2 | 18.66474 | 66.25 | 44 |
| SLC2A4 | 74.80081 | 67.83333 | 44 |
| TOP1 | 31.87312 | 66.83333 | 44 |
| PLK1 | 10.10208 | 64.33333 | 42 |
| MMP7 | 1.14619 | 66.16667 | 40 |
| FLT3 | 12.5219 | 65.83333 | 40 |
| NQO1 | 408.22198 | 66.33333 | 40 |
| ABCB1 | 19.69692 | 66.83333 | 40 |
| TOP2A | 243.71308 | 63.83333 | 38 |
| MMP3 | 5.73524 | 65.66667 | 38 |
| AURKB | 8.19819 | 62.83333 | 36 |
| ABCG2 | 171.8999 | 64.91667 | 36 |
| BAX | 0.38041 | 63.5 | 34 |
| ALOX5 | 23.54695 | 63 | 32 |
| NOX4 | 10.76352 | 64 | 32 |
| CD38 | 3.20695 | 63.33333 | 32 |
| BACE1 | 1.8957 | 63 | 30 |
| CYP1B1 | 70.87884 | 62.83333 | 30 |
| AKR1B1 | 320.03367 | 64 | 30 |
| PIK3CG | 6.15214 | 62.83333 | 28 |
| F2 | 61.4646 | 60.91667 | 28 |
| MMP13 | 0.16587 | 62.66667 | 28 |
| ACHE | 137.78998 | 62.08333 | 28 |
| ARG1 | 1.64908 | 59.91667 | 26 |
| PTGS1 | 21.34686 | 60.08333 | 26 |
| AXL | 0.19355 | 61.33333 | 24 |
| CA9 | 53.02675 | 61.66667 | 24 |
| CSNK2A1 | 7.05953 | 61.83333 | 24 |
| APEX1 | 87.20743 | 60.33333 | 22 |
| ALK | 0.29433 | 60 | 22 |
| PLA2G1B | 9.51933 | 58.58333 | 22 |
| CXCR1 | 1.37311 | 59.5 | 22 |
| AKR1C3 | 62.72868 | 56.83333 | 22 |
| TNKS | 13.09709 | 57.25 | 20 |
| MMP12 | 0.96464 | 59 | 20 |
| NEK2 | 10.62175 | 57.58333 | 18 |
| INSR | 0.97185 | 58.41667 | 18 |
| XDH | 80.86796 | 57.08333 | 18 |
| TYR | 5.91231 | 60 | 18 |
| ABCC1 | 0.37526 | 58.5 | 16 |
| TTR | 233.62719 | 57.16667 | 14 |
| ADORA1 | 0.66924 | 57 | 14 |
| ESRRA | 0.15385 | 57.25 | 12 |
| HSD17B1 | 3.85735 | 49.5 | 12 |
| ADORA2A | 0 | 55.75 | 12 |
| PIM1 | 0 | 54.66667 | 10 |
| DAPK1 | 0.27595 | 55.16667 | 10 |
| TCF7 | 0.1 | 54.91667 | 10 |
| CA2 | 36.32787 | 55.41667 | 10 |
| ALOX12 | 1.15235 | 50.91667 | 10 |
| PKN1 | 0.77357 | 55.75 | 10 |
| AKR1C1 | 2.32117 | 47.08333 | 10 |
| GLO1 | 30.50162 | 45.83333 | 10 |
| TNKS2 | 0.25 | 52.58333 | 8 |
| CD36 | 0.22619 | 53.66667 | 8 |
| CAMK2B | 0.7091 | 53.08333 | 8 |
| AKR1B10 | 6.69779 | 45.75 | 8 |
| MAOA | 25.07078 | 48.41667 | 8 |
| PTPRS | 0 | 53.83333 | 6 |
| EIF6 | 0 | 53 | 6 |
| CA4 | 0.55023 | 45.83333 | 6 |
| CA1 | 7.23016 | 44.66667 | 6 |
| DRD4 | 5.19528 | 50 | 4 |
| AVPR2 | 0 | 47.25 | 4 |
| AKR1A1 | 2.61461 | 43.08333 | 4 |
| SLC22A12 | 2.09875 | 43.66667 | 4 |
| NUAK1 | 0 | 51.91667 | 2 |
| NEK6 | 0 | 40.5 | 2 |
| DIO1 | 0 | 38.16667 | 2 |
| GPR35 | 0 | 42.58333 | 2 |

**Supplemental Table 3. The PPI network characteristics of the 91 intersection targets with the STRING web tool for direct physical interactions..**

| Target | Betweenness | Closeness | Degree |
| --- | --- | --- | --- |
| TP53 | 0.284978 | 0.560811 | 66 |
| HSP90AA1 | 0.147403 | 0.557047 | 60 |
| EGFR | 0.118749 | 0.522013 | 50 |
| SRC | 0.108422 | 0.528662 | 50 |
| CTNNB1 | 0.107935 | 0.482558 | 42 |
| PIK3R1 | 0.050816 | 0.506098 | 40 |
| JUN | 0.089581 | 0.509202 | 38 |
| AKT1 | 0.031737 | 0.461111 | 34 |
| CDK1 | 0.048942 | 0.50303 | 34 |
| RELA | 0.045051 | 0.471591 | 32 |
| AR | 0.022435 | 0.488235 | 26 |
| CDKN1A | 0.013662 | 0.471591 | 26 |
| GSK3B | 0.016491 | 0.491124 | 24 |
| CDK2 | 0.008559 | 0.458564 | 24 |
| CASP3 | 0.065958 | 0.439153 | 22 |
| CCND1 | 0.005602 | 0.419192 | 22 |
| MAPT | 0.036497 | 0.451087 | 20 |
| KDR | 0.006013 | 0.432292 | 20 |
| PTK2 | 0.005706 | 0.461111 | 20 |
| IGF1R | 0.003768 | 0.432292 | 18 |
| MET | 0.005154 | 0.432292 | 18 |
| APP | 0.037405 | 0.423469 | 16 |
| SYK | 0.002326 | 0.370536 | 16 |
| PARP1 | 0.008071 | 0.430052 | 16 |
| CDK6 | 8.61E-04 | 0.391509 | 16 |
| CDK4 | 0.002827 | 0.430052 | 16 |
| PLK1 | 0.007299 | 0.448649 | 16 |
| ESR2 | 0.003386 | 0.458564 | 16 |
| CSNK2A1 | 0.002304 | 0.448649 | 14 |
| CREB1 | 0.004802 | 0.432292 | 14 |
| AXL | 0.001081 | 0.408867 | 14 |
| CASP8 | 0.015514 | 0.42132 | 14 |
| CCND2 | 4.31E-04 | 0.384259 | 14 |
| TOP2A | 0.004169 | 0.419192 | 14 |
| PPARG | 0.028885 | 0.448649 | 14 |
| MCL1 | 0.050917 | 0.402913 | 12 |
| IL1B | 0.022205 | 0.404878 | 12 |
| MMP7 | 0.046779 | 0.382488 | 12 |
| TNF | 0.005122 | 0.402913 | 10 |
| CDC25C | 0.003111 | 0.395238 | 10 |
| CXCL8 | 0.015632 | 0.395238 | 10 |
| IL6 | 0.010367 | 0.382488 | 10 |
| MMP9 | 0.035189 | 0.356223 | 10 |
| ACHE | 0.027802 | 0.330677 | 8 |
| TERT | 0.001404 | 0.393365 | 8 |
| PIK3CG | 0 | 0.36087 | 8 |
| MMP2 | 0.015351 | 0.382488 | 8 |
| PTGS2 | 0.026098 | 0.425641 | 8 |
| CASP9 | 0.003206 | 0.406863 | 8 |
| TOP1 | 2.41E-04 | 0.400966 | 8 |
| ADORA2A | 0.025608 | 0.313208 | 6 |
| AHR | 3.36E-04 | 0.393365 | 6 |
| PKN1 | 4.75E-04 | 0.353191 | 6 |
| APEX1 | 1.16E-04 | 0.375566 | 6 |
| AURKB | 0 | 0.410891 | 6 |
| TCF7 | 0.002806 | 0.391509 | 6 |
| IL4 | 2.70E-04 | 0.301818 | 6 |
| CXCR1 | 2.70E-04 | 0.301818 | 6 |
| FLT3 | 0 | 0.351695 | 6 |
| INSR | 5.12E-04 | 0.397129 | 6 |
| AKR1C3 | 1 | 1 | 4 |
| NOS2 | 0 | 0.372197 | 4 |
| ALK | 0 | 0.350211 | 4 |
| TTR | 0 | 0.357759 | 4 |
| BACE1 | 0.001036 | 0.318008 | 4 |
| BAX | 0 | 0.370536 | 4 |
| CA9 | 0.024096 | 0.329365 | 4 |
| CD36 | 0 | 0.348739 | 4 |
| CD40LG | 0.001177 | 0.368889 | 4 |
| PIM1 | 0 | 0.384259 | 4 |
| MMP3 | 0 | 0.294326 | 4 |
| NQO1 | 0 | 0.372197 | 4 |
| XDH | 0 | 0.249249 | 2 |
| ADORA1 | 0 | 0.239193 | 2 |
| AKR1B1 | 0 | 1 | 2 |
| AKR1B10 | 0 | 1 | 2 |
| AKR1C1 | 0 | 0.666667 | 2 |
| HSD17B1 | 0 | 0.666667 | 2 |
| CA1 | 0 | 1 | 2 |
| CA2 | 0 | 1 | 2 |
| CA4 | 0 | 0.248503 | 2 |
| NEK2 | 0 | 0.326772 | 2 |
| DAPK1 | 0 | 0.36087 | 2 |
| ESRRA | 0 | 0.310861 | 2 |
| TNKS | 0 | 0.288194 | 2 |
| TNKS2 | 0 | 0.288194 | 2 |
| MMP12 | 0 | 0.277592 | 2 |
| MPO | 0 | 0.263492 | 2 |
| NOX4 | 0 | 0.34728 | 2 |
| NUAK1 | 0 | 0.36087 | 2 |
| PTGS1 | 0 | 0.299639 | 2 |

**Supplemental Table 4. The PPI network characteristics of the 123 intersection targets with the InAct tool for protein and protein interactions.**

| Target | Betweenness | Closeness | Degree |
| --- | --- | --- | --- |
| APP | 0.312674 | 0.473958 | 329 |
| P05067-PRO_0000000092 | 0.012256 | 0.322695 | 176 |
| EGFR | 0.125389 | 0.464286 | 160 |
| TP53 | 0.090576 | 0.419355 | 127 |
| CDKN1A | 0.025028 | 0.385593 | 104 |
| CCND1 | 0.029097 | 0.376033 | 84 |
| CTNNB1 | 0.101521 | 0.429245 | 80 |
| SRC | 0.075606 | 0.425234 | 80 |
| CDK4 | 0.012619 | 0.344697 | 77 |
| BAX | 0.105345 | 0.348659 | 75 |
| P05067-PRO_0000000093 | 0.017464 | 0.33829 | 72 |
| GSK3B | 0.118201 | 0.421296 | 71 |
| JUN | 0.107765 | 0.455 | 70 |
| HSP90AA1 | 0.116458 | 0.466667 | 69 |
| PIK3R1 | 0.036225 | 0.374486 | 65 |
| SYK | 0.008286 | 0.382353 | 56 |
| AR | 0.030746 | 0.408072 | 55 |
| CDK2 | 0.032973 | 0.376033 | 54 |
| MET | 0.03381 | 0.408072 | 52 |
| CDK1 | 0.083883 | 0.429245 | 45 |
| MAPT | 0.062691 | 0.415525 | 42 |
| AKT1 | 0.07924 | 0.419355 | 41 |
| CCND2 | 0.002696 | 0.299342 | 41 |
| PTK2 | 0.007871 | 0.365462 | 40 |
| IGF1R | 0.005754 | 0.355469 | 39 |
| PLK1 | 0.012619 | 0.379167 | 39 |
| MAPT | 0.030443 | 0.411765 | 38 |
| FLT3 | 9.33E-04 | 0.320423 | 35 |
| TTR | 0.00333 | 0.334559 | 35 |
| PARP1 | 0.011917 | 0.379167 | 33 |
| CASP3 | 0.060604 | 0.415525 | 33 |
| CSNK2A1 | 0.054403 | 0.408072 | 32 |
| ALK | 0 | 0.273273 | 27 |
| KDR | 0.00686 | 0.340824 | 25 |
| ADORA2A | 0 | 1 | 25 |
| CASP8 | 0.002513 | 0.366935 | 24 |
| APP | 0.021978 | 0.251381 | 22 |
| ESR2 | 0 | 0.337037 | 21 |
| ABCG2 | 1 | 1 | 21 |
| PIK3R1 | 0.007236 | 0.400881 | 20 |
| RELA | 0.003649 | 0.379167 | 19 |
| CDK6 | 0.009107 | 0.334559 | 18 |
| TOP2A | 0.005149 | 0.355469 | 18 |
| DAPK1 | 0 | 0.243316 | 18 |
| DRD4 | 0 | 0.322695 | 17 |
| CDC25C | 0.001071 | 0.348659 | 16 |
| CREB1 | 0 | 0.32852 | 16 |
| MMP2 | 0.019409 | 0.377593 | 16 |
| BACE1 | 0.043468 | 0.332117 | 16 |
| MCL1 | 0.086203 | 0.265306 | 15 |
| AXL | 7.48E-04 | 0.358268 | 14 |
| TOP1 | 7.57E-04 | 0.354086 | 14 |
| MMP9 | 0 | 0.260745 | 14 |
| TNF | 0.043468 | 0.327338 | 14 |
| INSR | 0 | 0.306397 | 13 |
| APEX1 | 0.004559 | 0.354086 | 13 |
| TERT | 2.34E-04 | 0.315972 | 13 |
| AURKB | 0.013303 | 0.358268 | 13 |
| NQO1 | 0 | 0 | 12 |
| GSK3B | 0 | 0.358268 | 10 |
| PIK3CG | 0 | 0.322695 | 10 |
| CASP9 | 0 | 0.294498 | 10 |
| CD36 | 0.001343 | 0.33829 | 10 |
| INSR | 0 | 0 | 10 |
| CASP9 | 0 | 0.294498 | 9 |
| CASP8 | 0 | 1 | 9 |
| RELA | 2.20E-04 | 0.327338 | 8 |
| ABCG2 | 0 | 0 | 8 |
| EIF6 | 1.63E-04 | 0.326165 | 7 |
| PIM1 | 0 | 0.666667 | 7 |
| CASP8 | 0 | 1 | 7 |
| NUAK1 | 0 | 0.296417 | 6 |
| PKN1 | 0.002871 | 0.356863 | 6 |
| PPARG | 0 | 0 | 6 |
| CAMK2B | 0 | 0 | 6 |
| PIM1 | 0 | 0.666667 | 6 |
| PPARG | 0 | 0 | 6 |
| PIM1 | 0.021978 | 0.320423 | 5 |
| TCF7 | 0 | 0.301325 | 5 |
| MCL1 | 0 | 1 | 5 |
| MCL1 | 0 | 1 | 5 |
| ADORA1 | 0 | 1 | 5 |
| CA2 | 4.21E-04 | 0.322695 | 4 |
| TNKS2 | 0.021978 | 0.211137 | 4 |
| MAPT | 0 | 0.320423 | 4 |
| P06213-PRO_0000016689 | 0 | 0.274096 | 4 |
| HSP90AA1 | 1.98E-04 | 0.274096 | 4 |
| PTPRS | 0.021978 | 0.248634 | 4 |
| HSD17B1 | 0 | 1 | 4 |
| ALOX5 | 0 | 1 | 4 |
| CDK4 | 1.67E-04 | 0.275758 | 4 |
| F2 | 0 | 0.322695 | 4 |
| PTPRS | 0 | 0 | 4 |
| NEK2 | 0 | 0 | 4 |
| TNKS | 0 | 0 | 4 |
| SYK | 0 | 0.322695 | 4 |
| PTGS2 | 0 | 0 | 4 |
| CA9 | 0 | 0 | 4 |
| INSR | 0 | 0 | 4 |
| ACHE | 0 | 0 | 4 |
| P01375-PRO_0000034424 | 0 | 0 | 4 |
| BACE1 | 0 | 0.322695 | 3 |
| Q14790-PRO_0000004631 | 0 | 1 | 3 |
| Q14790-PRO_0000004629 | 0 | 1 | 3 |
| AHR | 0 | 0.301325 | 2 |
| BAX | 0 | 0.210162 | 2 |
| ARG1 | 0 | 0.210162 | 2 |
| CXCR1 | 0 | 0.301325 | 2 |
| TOP2A | 0 | 0.290735 | 2 |
| HSP90AA1 | 0 | 0.290735 | 2 |
| MMP3 | 0 | 0.199561 | 2 |
| NANOS2 | 0 | 0.174664 | 2 |
| AKR1C1 | 0 | 1 | 2 |
| AKR1C3 | 0 | 1 | 2 |
| MPO | 0 | 0.297386 | 2 |
| ARG1 | 0 | 0.297386 | 2 |
| MAPT | 0 | 0.299342 | 2 |
| MAPT | 0 | 0.299342 | 2 |
| ABCB1 | 0 | 0.201327 | 2 |
| P42574-PRO_0000004571 | 0 | 1 | 2 |
| P42574-PRO_0000004572 | 0 | 1 | 2 |
| CAMK2B | 0 | 0.294498 | 2 |
| MAPT | 0 | 0.294498 | 2 |

**Supplemental Table 5. GO enrichment analysis using the 118 intersection targets.**

| Category | Term | Count | P value |
| --- | --- | --- | --- |
| BP | GO: 0006979～response to oxidative stress | 33 | 7.54E-26 |
|  | GO: 1901652～response to peptide | 31 | 4.4E-22 |
|  | GO: 0009636～response to toxic substance | 31 | 7.1E-22 |
|  | GO: 0034599～cellular response to oxidative stress | 28 | 9.73E-25 |
|  | GO: 0071900～regulation of protein serine/threonine kinase activity | 25 | 6.73E-16 |
|  | GO: 1901653～cellular response to peptide | 24 | 1.41E-17 |
|  | GO: 0043410～positive regulation of MAPK cascade | 24 | 1.69E-14 |
|  | GO: 0000302～response to reactive oxygen species | 23 | 6.20E-21 |
|  | GO: 0072593～reactive oxygen species metabolic process | 23 | 2.20E-19 |
|  | GO: 0035690～cellular response to drug | 23 | 1.62E-16 |
| CC | GO: 0044454～nuclear chromosome part | 16 | 4.30E-08 |
|  | GO: 0045121～membrane raft | 14 | 9.55E-09 |
|  | GO: 0098857～membrane microdomain | 14 | 9.55E-09 |
|  | GO: 0098589～membrane region | 14 | 1.55E-08 |
|  | GO: 0061695～transferase complex, transferring phosphorus-containing groups | 12 | 1.66E-09 |
|  | GO: 0043025～neuronal cell body | 12 | 3.06E-05 |
|  | GO: 0005667～transcription factor complex | 11 | 1.15E-06 |
|  | GO: 0045177～apical part of cell | 11 | 2.02E-05 |
|  | GO: 0005813～centrosome | 11 | 0.00020479 |
|  | GO: 0000785～chromatin | 11 | 0.000329147 |
| MF | GO: 0004674～protein serine/threonine kinase activity | 19 | 1.03E-11 |
|  | GO: 0046982～protein heterodimerization activity | 16 | 3.43E-07 |
|  | GO: 0003682～chromatin binding | 13 | 1.10E-05 |
|  | GO: 0004713～protein tyrosine kinase activity | 11 | 7.03E-10 |
|  | GO: 0019902～phosphatase activity | 11 | 4.24E-08 |
|  | GO: 0004175～endopeptidase activity | 11 | 1.52E-05 |
|  | GO: 0030545～receptor regulator activity | 11 | 0.000184919 |
|  | GO: 0048037～cofactor binding | 11 | 0.000239776 |
|  | GO: 0019903～protein phosphatase binding | 10 | 2.75E-08 |
|  | GO: 0005126～cytokine receptor binding | 10 | 2.07E-06 |

**Supplemental Table 6. GO enrichment analysis using the top 20 hub genes.**

| Category | Term | Count | P value |
| --- | --- | --- | --- |
| BP | GO:0062197～cellular response to chemical stress | 12 | 1.72E-16 |
|  | GO: 0006979～response to oxidative stress | 12 | 2.36E-15 |
|  | GO: 0009410～response to xenobiotic stimulus | 11 | 1.58E-13 |
|  | GO: 0034599～cellular response to oxidative stress | 10 | 1.16E-13 |
|  | GO: 1901214～regulation of neuron death | 10 | 3.86E-13 |
|  | GO: 0070997～neuron death | 10 | 1.43E-12 |
|  | GO: 2001233～regulation of apoptotic signaling pathway | 10 | 1.77E-12 |
|  | GO: 0048660～regulation of smooth muscle cell proliferation | 9 | 7.55E-14 |
|  | GO: 0048659～smooth muscle cell proliferation | 9 | 9.27E-14 |
|  | GO: 2001234～negative regulation of apoptotic signaling pathway | 9 | 9.63E-13 |
|  | GO: 0033002～muscle cell proliferation | 9 | 1.75E-12 |
|  | GO: 0032496～response to lipopolysaccharide | 9 | 3.27E-11 |
|  | GO: 0002237～response to molecule of bacterial origin | 9 | 5.53E-11 |
|  | GO: 0010038～response to metal ion | 9 | 5.66E-11 |
|  | GO: 0030099～myeloid cell differentiation | 9 | 1.92E-10 |
|  | GO: 0048732～gland development | 9 | 2.89E-10 |
|  | GO: 1903829～positive regulation of protein localization | 9 | 4.89E-10 |
|  | GO: 0019221～cytokine-mediated signaling pathway | 9 | 7.59E-10 |
|  | GO: 0002573～myeloid leukocyte differentiation | 8 | 4.80E-11 |
|  | GO: 0071216～cellular response to biotic stimulus | 8 | 1.60E-10 |
|  | GO: 0097193～intrinsic apoptotic signaling pathway | 8 | 4.54E-10 |
|  | GO: 0018105～peptidyl-serine phosphorylation | 8 | 5.58E-10 |
|  | GO: 0018209～peptidyl-serine modification | 8 | 8.73E-10 |
|  | GO: 0071900～regulation of protein serine/threonine kinase activity | 8 | 2.26E-09 |
|  | GO: 0001503～ossification | 8 | 7.37E-09 |
| CC | GO: 0045121～membrane raft | 7 | 2.05E-08 |
|  | GO: 0098857～membrane microdomain | 7 | 2.10E-08 |
|  | GO: 0017053～transcription repressor complex | 4 | 1.09E-06 |
|  | GO: 0090575～RNA polymerase II transcription regulator complex | 4 | 0.0001152 |
|  | GO: 0031253～cell projection membrane | 4 | 0.0003881 |
|  | GO: 0098978～glutamatergic synapse | 4 | 0.0007119 |
|  | GO: 0031252～cell leading edge | 4 | 0.000793 |
|  | GO: 0043025～neuronal cell body | 4 | 0.0014876 |
|  | GO: 0000791～euchromatin | 3 | 3.03E-05 |
|  | GO: 0005901～caveola | 3 | 8.02E-05 |
|  | GO: 0044853～plasma membrane raft | 3 | 0.0002058 |
|  | GO: 0019897～extrinsic component of plasma membrane | 3 | 0.0004344 |
|  | GO: 0016323～basolateral plasma membrane | 3 | 0.001668 |
|  | GO: 0031968～organelle outer membrane | 3 | 0.001709 |
|  | GO: 0019867～outer membrane | 3 | 0.0017508 |
|  | GO: 0009925～basal plasma membrane | 3 | 0.0024032 |
|  | GO: 0045178～basal part of cell | 3 | 0.0029216 |
|  | GO: 0019898～extrinsic component of membrane | 3 | 0.0030092 |
|  | GO: 0031965～nuclear membrane | 3 | 0.0036354 |
|  | GO: 0005925～focal adhesion | 3 | 0.0087034 |
|  | GO: 0030055～cell-substrate junction | 3 | 0.0092777 |
|  | GO: 0005635～nuclear envelope | 3 | 0.0130472 |
|  | GO: 0030877～beta-catenin destruction complex | 2 | 5.46E-05 |
|  | GO: 0044292～dendrite terminus | 2 | 7.73E-05 |
|  | GO: 1990909～Wnt signalosome | 2 | 7.73E-05 |
| MF | GO: 0061629～RNA polymerase II-specific DNA-binding transcription factor binding | 7 | 5.17E-08 |
|  | GO: 0140297～DNA-binding transcription factor binding | 7 | 4.47E-07 |
|  | GO: 0005126～cytokine receptor binding | 6 | 3.11E-07 |
|  | GO: 0031625～ubiquitin protein ligase binding | 6 | 5.88E-07 |
|  | GO: 0044389～ubiquitin-like protein ligase binding | 6 | 8.41E-07 |
|  | GO: 0002020～protease binding | 4 | 1.35E-05 |
|  | GO: 0019903～protein phosphatase binding | 4 | 1.42E-05 |
|  | GO: 0019902～phosphatase binding | 4 | 4.44E-05 |
|  | GO: 0005125～cytokine activity | 4 | 0.0001077 |
|  | GO: 0048018～receptor ligand activity | 4 | 0.0018173 |
|  | GO: 0016004～phospholipase activator activity | 3 | 1.68E-06 |
|  | GO: 0060229～lipase activator activity | 3 | 2.50E-06 |
|  | GO: 0097718～disordered domain specific binding | 3 | 7.70E-06 |
|  | GO: 0032813～tumor necrosis factor receptor superfamily binding | 3 | 1.85E-05 |
|  | GO: 0097110～scaffold protein binding | 3 | 4.83E-05 |
|  | GO: 0046332～SMAD binding | 3 | 7.67E-05 |
|  | GO: 0042826～histone deacetylase binding | 3 | 0.0003299 |
|  | GO: 0044325～transmembrane transporter binding | 3 | 0.0003535 |
|  | GO: 0070851～growth factor receptor binding | 3 | 0.0004216 |
|  | GO: 0016922～nuclear receptor binding | 3 | 0.0004307 |
|  | GO: 0045296～cadherin binding | 3 | 0.0053539 |
|  | GO: 0004674～protein serine/threonine kinase activity | 3 | 0.0101981 |
|  | GO: 0004175～endopeptidase activity | 3 | 0.0106647 |
|  | GO: 0001228～DNA-binding transcription activator activity, RNA polymerase II-specific | 3 | 0.0135701 |
|  | GO: 0001216～DNA-binding transcription activator activity | 3 | 0.0138833 |

**Supplemental Table 7. KEGG pathway enrichment analysis (top 20 with count) using the 118 intersection targets.**

| **Pathway** | **Genes** | **Count** | **P value** |
| --- | --- | --- | --- |
| PI3K-Akt signaling pathway | MET/GSK3B/EGFR/CREB1/CDK2/CDKN1A/PIK3R1/MCL1/IL6/PIK3CG/SYK/IGF1R/RELA/PKN1/INSR/AKT1/PTK2/CCND1/CDK4/IL4/TP53/CASP9/CDK6/HSP90AA1/KDR/FLT3/CCND2 | 27 | 8.31E-14 |
| Human cytomegalovirus infection | GSK3B/EGFR/CREB1/CDKN1A/PIK3R1/IL6/CASP3/RELA/AKT1/PTK2/TNF/PTGS2/CTNNB1/CCND1/CDK4/SRC/TP53/CASP9/IL1B/CASP8/CXCL8/CDK6/BAX | 23 | 1.56E-14 |
| Kaposi sarcoma-associated herpesvirus infection | GSK3B/CREB1/CDKN1A/PIK3R1/IL6/CASP3/PIK3CG/SYK/RELA/AKT1/JUN/PTGS2/CTNNB1/CCND1/CDK4/SRC/TP53/CASP9/CASP8/CXCL8/CDK6/BAX | 22 | 2.89E-15 |
| Human papillomavirus infection | GSK3B/EGFR/CREB1/CDK2/CDKN1A/PIK3R1/CASP3/RELA/AKT1/TCF7/PTK2/TNF/PTGS2/CTNNB1/CCND1/CDK4/TERT/TP53/CASP8/CDK6/CCND2/BAX | 22 | 3.36E-10 |
| Prostate cancer | GSK3B/MMP3/EGFR/CREB1/CDK2/CDKN1A/PIK3R1/IGF1R/RELA/AKT1/TCF7/AR/CTNNB1/CCND1/TP53/CASP9/HSP90AA1/MMP9 | 18 | 3.66E-16 |
| Measles | GSK3B/CDK2/PIK3R1/CSNK2A1/IL6/CASP3/RELA/AKT1/JUN/CCND1/CDK4/TP53/CASP9/IL1B/CASP8/CDK6/CCND2/BAX | 18 | 2.26E-13 |
| Epstein-Barr virus infection | CDK2/CDKN1A/PIK3R1/IL6/CASP3/SYK/RELA/AKT1/JUN/TNF/CCND1/CDK4/TP53/CASP9/CASP8/CDK6/CCND2/BAX | 18 | 1.44E-10 |
| Hepatitis C | GSK3B/EGFR/CDK2/CDKN1A/PIK3R1/CASP3/RELA/AKT1/TNF/CTNNB1/CCND1/CDK4/TP53/CASP9/CASP8/CDK6/BAX | 17 | 1.94E-11 |
| Hepatitis B | CREB1/CDK2/CDKN1A/PIK3R1/IL6/CASP3/RELA/AKT1/JUN/TNF/SRC/TP53/CASP9/CASP8/CXCL8/BAX/MMP9 | 17 | 3.97E-11 |
| Viral carcinogenesis | CDK1/CREB1/CDK2/CDKN1A/PIK3R1/CASP3/SYK/RELA/JUN/CCND1/CDK4/SRC/TP53/CASP8/CDK6/CCND2/BAX | 17 | 1.21E-09 |
| Proteoglycans in cancer | MMP2/MET/CAMK2B/EGFR/CDKN1A/PIK3R1/CASP3/IGF1R/AKT1/PTK2/TNF/CTNNB1/CCND1/SRC/TP53/KDR/MMP9 | 17 | 1.52E-09 |
| MicroRNAs in cancer | MET/EGFR/CDKN1A/PIK3R1/ABCB1/MCL1/CASP3/PTGS2/CCND1/TP53/ABCC1/CYP1B1/CDC25C/PIM1/CDK6/CCND2/MMP9 | 17 | 7.34E-07 |
| Hepatocellular carcinoma | MET/GSK3B/EGFR/CDKN1A/PIK3R1/NQO1/IGF1R/AKT1/TCF7/CTNNB1/CCND1/CDK4/TERT/TP53/CDK6/BAX | 16 | 6.76E-10 |
| Human T-cell leukemia virus 1 infection | MMP7/CREB1/CDK2/CDKN1A/PIK3R1/IL6/RELA/AKT1/JUN/TNF/CCND1/CDK4/TERT/TP53/CCND2/BAX | 16 | 3.19E-08 |
| Small cell lung cancer | NOS2/CDK2/CDKN1A/PIK3R1/CASP3/RELA/AKT1/PTK2/PTGS2/CCND1/CDK4/TP53/CASP9/CDK6/BAX | 15 | 9.37E-13 |
| IL-17 signaling pathway | GSK3B/MMP3/MMP13/IL6/CASP3/RELA/JUN/TNF/PTGS2/IL4/IL1B/CASP8/CXCL8/HSP90AA1/MMP9 | 15 | 1.11E-12 |
| Endocrine resistance | MMP2/EGFR/CDKN1A/PIK3R1/IGF1R/AKT1/JUN/PTK2/CCND1/CDK4/SRC/TP53/ESR2/BAX/MMP9 | 15 | 2.45E-12 |
| AGE-RAGE signaling pathway in diabetic complications | MMP2/PIK3R1/IL6/CASP3/RELA/AKT1/JUN/TNF/CCND1/CDK4/IL1B/CXCL8/PIM1/NOX4/BAX | 15 | 3.32E-12 |
| Fluid shear stress and atherosclerosis | MMP2/PIK3R1/NQO1/RELA/AKT1/JUN/PTK2/TNF/CTNNB1/SRC/TP53/IL1B/HSP90AA1/KDR/MMP9 | 15 | 4.15E-10 |
| Breast cancer | GSK3B/EGFR/CDKN1A/PIK3R1/IGF1R/AKT1/TCF7/JUN/CTNNB1/CCND1/CDK4/TP53/ESR2/CDK6/BAX | 15 | 9.20E-10 |

**Supplemental Table 8. KEGG pathway enrichment analysis (top 25 with count) using the top 20 hub genes.**

| **Pathway** | **Genes** | **Count** | **P value** |
| --- | --- | --- | --- |
| Human cytomegalovirus infection | TP53/AKT1/TNF/IL6/CASP3/CTNNB1/SRC/EGFR/IL1B/PTGS2/CCND1/CXCL8/CREB1/CASP8/GSK3B | 15 | 5.51E-20 |
| Pathways in cancer | TP53/AKT1/IL6/CASP3/CTNNB1/JUN/EGFR/HSP90AA1/PTGS2/CCND1/MMP9/PPARG/CXCL8/CASP8/GSK3B | 15 | 2.29E-14 |
| Kaposi sarcoma-associated herpesvirus infection | TP53/AKT1/IL6/CASP3/CTNNB1/JUN/SRC/PTGS2/CCND1/CXCL8/CREB1/CASP8/GSK3B | 13 | 2.95E-17 |
| IL-17 signaling pathway | TNF/IL6/CASP3/JUN/HSP90AA1/IL1B/PTGS2/MMP9/CXCL8/CASP8/GSK3B | 11 | 4.95E-17 |
| Hepatitis B | TP53/AKT1/TNF/IL6/CASP3/JUN/SRC/MMP9/CXCL8/CREB1/CASP8 | 11 | 2.70E-14 |
| Human papillomavirus infection | TP53/AKT1/TNF/CASP3/CTNNB1/EGFR/PTGS2/CCND1/CREB1/CASP8/GSK3B | 11 | 6.75E-11 |
| TNF signaling pathway | AKT1/TNF/IL6/CASP3/JUN/IL1B/PTGS2/MMP9/CREB1/CASP8 | 10 | 3.52E-14 |
| Prostate cancer | TP53/AKT1/CTNNB1/EGFR/HSP90AA1/CCND1/MMP9/CREB1/GSK3B | 9 | 6.43E-13 |
| Measles | TP53/AKT1/IL6/CASP3/JUN/IL1B/CCND1/CASP8/GSK3B | 9 | 1.64E-11 |
| Fluid shear stress and atherosclerosis | TP53/AKT1/TNF/CTNNB1/JUN/SRC/HSP90AA1/IL1B/MMP9 | 9 | 1.75E-11 |
| Non-alcoholic fatty liver disease (NAFLD) | AKT1/TNF/IL6/CASP3/JUN/IL1B/CXCL8/CASP8/GSK3B | 9 | 3.29E-11 |
| Hepatitis C | TP53/AKT1/TNF/CASP3/CTNNB1/EGFR/CCND1/CASP8/GSK3B | 9 | 4.70E-11 |
| Proteoglycans in cancer | TP53/AKT1/TNF/CASP3/CTNNB1/SRC/EGFR/CCND1/MMP9 | 9 | 5.54E-10 |
| PI3K-Akt signaling pathway | TP53/AKT1/IL6/EGFR/HSP90AA1/CCND1/CREB1/MCL1/GSK3B | 9 | 7.03E-08 |
| Colorectal cancer | TP53/AKT1/CASP3/CTNNB1/JUN/EGFR/CCND1/GSK3B | 8 | 1.58E-11 |
| AGE-RAGE signaling pathway in diabetic complications | AKT1/TNF/IL6/CASP3/JUN/IL1B/CCND1/CXCL8 | 8 | 5.44E-11 |
| C-type lectin receptor signaling pathway | AKT1/TNF/IL6/JUN/SRC/IL1B/PTGS2/CASP8 | 8 | 7.49E-11 |
| Yersinia infection | AKT1/TNF/IL6/JUN/SRC/IL1B/CXCL8/GSK3B | 8 | 2.39E-10 |
| Tuberculosis | AKT1/TNF/IL6/CASP3/SRC/IL1B/CREB1/CASP8 | 8 | 5.85E-09 |
| Epstein-Barr virus infection | TP53/AKT1/TNF/IL6/CASP3/JUN/CCND1/CASP8 | 8 | 1.46E-08 |
| Herpes simplex virus 1 infection | TP53/AKT1/TNF/IL6/CASP3/SRC/IL1B/CASP8 | 8 | 1.34E-05 |
| Endocrine resistance | TP53/AKT1/JUN/SRC/EGFR/CCND1/MMP9 | 7 | 2.44E-09 |
| Chagas disease (American trypanosomiasis) | AKT1/TNF/IL6/JUN/IL1B/CXCL8/CASP8 | 7 | 3.24E-09 |
| Toll-like receptor signaling pathway | AKT1/TNF/IL6/JUN/IL1B/CXCL8/CASP8 | 7 | 3.72E-09 |
| Apoptosis | TP53/AKT1/TNF/CASP3/JUN/CASP8/MCL1 | 7 | 2.44E-08 |
